# Genome Editing in Crop Plant Research—Alignment of Expectations and Current Developments

**DOI:** 10.3390/plants11020212

**Published:** 2022-01-14

**Authors:** Meike Hüdig, Natalie Laibach, Anke-Christiane Hein

**Affiliations:** 1 Molecular Plant Physiology Division, Institute of Molecular Physiology and Biotechnology of Plants, University of Bonn, Kirschallee 1, 53115 Bonn, Germany; 2 Centre for Research in Agricultural Genomics (CRAG), Edifici CRAG—Campus UAB, 08193 Cerdanyola del Vallès, Spain; 3 Federal Agency for Nature Conservation, Assessment of Genetically Modified Organisms, Konstantinstraße 110, 53179 Bonn, Germany; anke-christiane.hein@bfn.de

**Keywords:** genome editing, crops, CRISPR, sustainable development goals

## Abstract

The rapid development of genome editing and other new genomic techniques (NGT) has evoked manifold expectations on purposes of the application of these techniques to crop plants. In this study, we identify and align these expectations with current scientific development. We apply a semi-quantitative text analysis approach on political, economic, and scientific opinion papers to disentangle and extract expectations towards the application of NGT-based plants. Using the sustainable development goals (SDG) of the 2030 agenda as categories, we identify contributions to food security or adaptation to climatic changes as the most frequently mentioned expectations, accompanied by the notion of sustainable agriculture and food systems. We then link SDG with relevant plant traits and review existing research and commercial field trials for genome-edited crop plants. For a detailed analysis we pick as representative traits drought tolerance and resistance against fungal pathogens. Diverse genetic setscrews for both traits have been identified, modified, and tested under laboratory conditions, although there are only a few in the field. All in all, NGT-plants that can withstand more than one stressor or different environments are not documented in advanced development states. We further conclude that developing new plants with modified traits will not be sufficient to reach food security or adaption to climatic changes in a short time frame. Further scientific development of sustainable agricultural systems will need to play an important role to tackle SDG challenges, as well.

## 1. Introduction

In the last decade, plant biotechnology has undergone a profound development. Particularly, genome-editing techniques based on CRISPR/Cas (clustered regularly interspaced palindromic repeats) have rapidly advanced research in plant biology. These and other new genomic techniques (NGT) have evolved many different facets compared to its beginnings several years ago. Besides site-directed nucleases, such as CRISPR/Cas, TALEN (transcription activator-like effector nuclease) or zinc finger nucleases, other techniques, including ODM (oligonucleotide directed mutagenesis), RdDm (RNA dependent DNA methylation), cis- and intragenesis, or trans-grafting or agroinfiltration (“sensu stricto”), were broadly applied in plant development after Directive 2001/18/EC entered the force [1]. Currently, NGT mostly refer to site-directed nucleases (SDN) and deviated techniques [2]. SDN are often distinguished into categories for regulatory reasons [3]. SDN1 triggers DNA repair via non-homologous end-joining (NHEJ) after the nucleolytic cut, which may lead to insertion or deletion of random sequences at the intersection, whereas SDN2 alters short nucleotide sequences or introduces point mutations via homology directed repair (HDR). SDN1 and SDN2 are mostly referred to in the term genome editing. SDN3 allows for the insertion of foreign DNA or complete exchanges of entire gene and cis-regulatory elements via HDR. Hence, SDN3 can include transgenesis, but in contrast to classical engineering, at pre-defined sequence positions [4].

New genomic techniques increasingly allow for far-reaching alterations in plant genomes that are not comparable in kind, number, and efficiency to classical breeding or transgenesis techniques, such as agrobacterium-mediated or ballistic transformation [5,6]. Using recently developed Cas-related systems, double-strand breaks are often dispensable for techniques like base editing, prime editing, or site-directed RNA editing modifying only one or several nucleotides [2,7,8]. Most noteworthy, genomic regions with low local recombination rates, which can now be accessed as CRISPR/Cas, may bypass certain genome repair mechanisms [9,10,11].

Both the general possibilities of CRISPR/Cas in plant breeding and more specified topics such as off-target effects and specificity improvements have been extensively reviewed [12,13,14,15,16]. In addition to (gene-specific) mutagenesis in coding sections, methods for specific editing or the targeted exchange of specific, individual nucleobases (base editing) have been developed, which enable a more specific change in gene function, e.g., decreased or increased enzyme activity [14,17,18], for example, when applied in watermelon [19]. This is possible by fusing the Cas enzyme with a deaminase. In addition, the editing of cis-regulatory areas or the epigenetic modification by inactive nucleases (e.g., dCas9), which is possible through fusion with effector proteins, such as (de-)methyltransferases, enables a more precise control of gene expression [18,20,21,22]. These techniques enable, for instance, the altered expression of numerous downstream genes by introducing epigenetic changes through, e.g., DNA methylation patterns [23,24,25] or the swapping of cis-regulatory elements and binding sites of transcription factors [26]. In the latter case, a certain piece of DNA (entire, partial gene, or promoter sequences) in the plant’s genome can be exchanged for another defined DNA fragment at a defined location [24]. This application has so far been rare, but has been used for the introduction of a herbicide resistance gene OsEPSPS in rice [27] or a stronger promoter in maize [26].

Moreover, editing enzymes more suitable for plant cells, such as Cpf1 and Cas12a, [18] as well as the adaptation of guide RNAs by preparatory bioinformatics searches for potential off-target sequences and a subsequently optimised design of guide RNAs, is improving specificity [15,28,29]. However, unintended effects, such as off-target edits, cis bystander-effects that may cause unintended rearrangements, exon skipping, or single-strand nicking remain challenging [30]. It is noteworthy that commonly applied analytical methods and bioinformatic approaches mostly cover sequence-specific off-target effects but neglect most non-specific bystander effects. Yet, more efficient methods for genome sequencing and long range PCR are currently being further developed [15,31,32]. 

However, a certain imprecision in specificity can be used to intentionally modify more than one allele, which is especially advantageous for polyploid crops. Multi-allele modifications through NGT have been successfully applied for cotton, tomato, rice, poplar, and ornamental plants [33]. Such multiplexing can be also applied using different guide RNAs, thus targeting different genes of one pathway or even functionally and locally unrelated genes in a row by using different guide RNAs [26,28,34,35,36,37,38,39,40,41,42,43]. These attempts have been used for de novo domestication of wild tomato and can be used to introduce domestication traits into resilient wild relatives of other crops [37,44,45]. Robust new techniques accelerate velocity and quality of basic plant research when multiple simultaneous experiments are conducted. This was illustrated by knockout studies, such as the deletion of 57 genes to elucidate pathogen resistance gene functions in rice mutant lines [46]. Once a desired genetic modification has been identified, it can be theoretically introduced into another plant within a reduced time frame compared to breeding or classical genetic engineering techniques, or transferred between plant species of a similar genetic background [47,48]. However, not all techniques are stably applicable for all purposes or have been established in all relevant plant species, e.g., due to the lack of efficient transformation methods for elite lines or entire crop species [6,40].

Despite the rapid development of editing techniques, certain general challenges remain on the technical level. For example, recombination effects or integration of editing components via classical transformation methods or effects from in vitro cultivation, such as somaclonal variation cannot, thus far, be entirely excluded [49]. Adapted transformation methods and transient editing tools aim to circumvent these effects. Methods, such as in vitro assembled ribonucleoprotein particles (RNPs), virus-mediated delivery of RNA templates, nanoparticles, or agrobacterium-mediated vesicle delivery, have been established for several plant species [49,50]; adapted guide RNA usage [40] and approaches, such as de novo meristemisation, aim to avoid tissue culture steps in dicotyledonous plants [51].

Therefore, understanding how exactly a modification finally manifests in the plant’s phenotype depends on many factors and can, until now, hardly be predicted [6,52]. Modified plants will still have to be based on corresponding studies for the years to come, taking the native context of the genetic information into account and evaluating the intended growing environmental conditions. This is especially valid for complex traits, such as abiotic stress tolerance, but also for combinations of traits that are increasingly demanded by stakeholders and currently developed in transgenic plant varieties intended for market release. However, the actual phenotypic and ecological outcomes of complex traits and combinations, be it changes in metabolism, tolerance to abiotic stress, or resistance to predators, pathogens, and herbicides, may vary for every new trait and plant variety, and can most likely only be assessed empirically [6,53].

While these downstream developments are difficult to predict and come with their own challenges, the rate of development and findings in basic research has often evoked expectations in new genomic techniques in the field of crop sciences. As with classical genetic engineering, expectations on the part of policy-makers, stakeholders, and the scientific community are highly diverse and often linked to general agricultural aims in the near future (e.g., until 2030), such as pronounced in the European Farm-to-Fork or biodiversity strategies [54,55]. In addition, the framework of the UN Sustainable Development Goals (SDG, 2030 Agenda for Sustainable Development [56]) calls for action to improve sustainability and resilience of agriculture while providing food security in both quality and quantity. This sets high stakes for agriculture in general but also for plant breeding in particular. 

Here, we aim to identify expectations by literature analyses of statements of German, European, and international organisations. We compare these stakeholder expectations to the two state-of-the-art exemplary research areas, resilience to drought and resistance to filamentous pathogens. Based on preceding studies, we give an overview on developmental status of the research areas and discuss their potential contribution to long-term agricultural aims.

## 2. Results

### 2.1. Expectations of Genome Editing in Crop Plant Development Are Diverse, of Abstract Nature, and Differ among Stakeholders

Future plant development options and therein NGT are, in sight of upcoming challenges for agriculture such as climate change adaptation [23,57,58], actively discussed. This discussion is reflected in written expectations within documents from scientists and politicians (see Table S1 for documents used). To systematically assess those expectations towards new genetic engineering, apart from specialist publications, statements from various interest groups and political documents were screened and categorised by applying computer-assisted semi-quantitative content analysis [59,60]. As a framework for deductive coding, the UN Sustainable Development Goals were used. 

**Figure 1:**
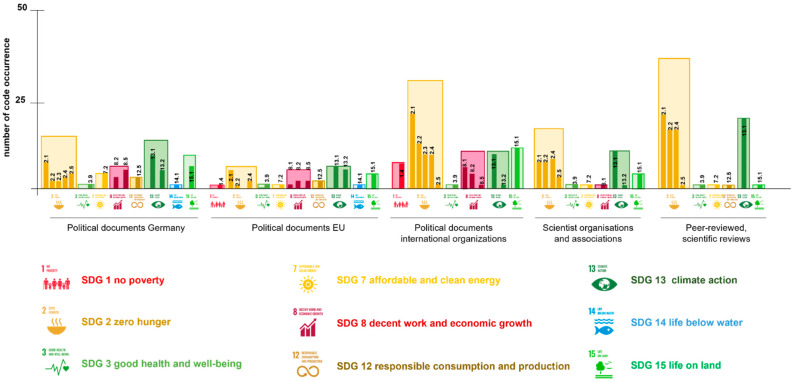
Numbers of expectations coded using SDG per analysed documents. Political documents Germany; political documents EU; political documents international organisations; scientist organisations and associations; Pub: peer-reviewed, scientific reviews. Sustainable Development Goals (SDG): SDG 1: no poverty; SDG 1.4: equal access to resources; SDG 2: zero hunger; SDG 2.1: nutrition quantity and food security; SDG 2.3: improve smallholder situation; SDG 2.5: ensure agricultural genetic diversity; SDG2.4: sustainable and resilient agriculture; SDG 2.2: nutrition improvement; SDG 3: good health and well-being; SDG3.9: reduce illness from contamination/allergy; SDG 7: affordable and clean energy; SDG 7.2: renewable energy; SDG 8: decent work and economic growth; SDG 8.1: inclusive economic growth; SDG 8.5: employment; SDG 8.2: innovation; SDG 12: responsible consumption and economic growth; SDG 12.5: reduce waste, recycle; SDG 13: climate action; SDG 13.1: adaptation actions; SDG 13.2: mitigation actions; SDG 14: life below water; SDG 14.1: avoid pollution and overnutrition; SDG 15: life on land; SDG 15.1: ecosystem conservation [56].

Overall, expectations towards plant breeding relate mainly to food security (SDG 2—100 codings). More specifically, the subcategories of quantity (SDG 2.1, 58 codings) and quality of the diet (SDG 2.2, 36 codings), and sustainable and resilient agriculture (SDG 2.4, 38 codings) are often mentioned (Figure 1, Table 1). Adaptations to climatic changes (SDG 13, 52 codings), such as extreme temperatures and drought, etc., are also central, whereas the mitigation of climate change plays a rather subordinate role or is partly reflected in more general statements coded with SDG 2.4. However, the protection of terrestrial ecosystems (SDG 15, 27 codings) is nonetheless a central point in the expectations. In connection with SDG 2.3 (improve smallholder situation), the objective SDG 8 (decent work and economic growth, 22 codings), which also occurs frequently, should be mentioned. The coded expectations are related to the income security of farmers of all kinds (smallholder to large-scale industrial production), but also relate to job security and growth through innovation (SDG 8.2, 10 codings). The latter is also often named in the context of the development of new, bio-based technologies and renewable energies (SDG 7, 7 codings). Albeit, these expectations, similarly to the protection of marine ecosystems (SDG 14, 2 codings), play a smaller role compared to food security or climate change adaptations.

The expectations coded by SDG in connection with agriculture are prioritised differently in the analysed document groups. The focus in scientific reviews or in statements by scientific organisations is primarily on security and quality of nutrition, as well as on adaptation to climate change with more resilient and sustainable agriculture (see Figure 1, Table 1). Points, such as the economic improvement or the protection of biodiversity, are not found at all in the documents examined or are rather subordinate with 1–4 mentions. Similarly, mainly economic goals, such as SDG 1 (end poverty) and SDG 8 (sustainable economic growth), are mentioned to a lesser extent in documents of the OECD and economy-related documents, in contrast to 19 codings in political papers. The central goals in primarily political documents, which are reflected, e.g., by the FAO (Food and Agriculture Organization) of the United Nations [61,62], are above all the security and quality of nutrition (SDG 2). Agriculture that is both sustainable and resilient to climate change and other challenges, such as pathogen resistance, is also of central importance throughout the political statements, e.g., by the German Federal Ministry of Food and Agriculture [63]. The preservation and increase in genetic diversity in agriculture (SDG 2.5) is an explicitly mentioned objective, which also plays a larger role in political and economic documents relative to the number of documents (seven out of a total of nine mentions). The use of NGT is also considered for a future, integrated agricultural practice, e.g., as an instrument as part of a “Climate Smart Agriculture” [61,64]. Due to the expectation that NGT can provide (i) a stable yield, (ii) an increased resistance to plant diseases and climatic conditions as well as (iii) a more accessible and faster breeding, this concept also aims at the economic security of small farmers [62,63].

Nutrient content and the quality of crops are further important aspects of plant traits potentially achievable with NGT (see Figure 1, SDG 2.2). In the analysed documents of international organisations, this goal often occurs with 12 mentions compared to other political documents (1–5 mentions). With 16 codings, this goal is mentioned with similar abundance in scientific publications. 

Finally, it should be pointed out that there was mostly no differentiation between NGT, other breeding methods, or general agricultural practice in the political documents—i.e., general goals were often described and NGT mentioned elsewhere as means to achieve them or even subsumed under new biotechnological or technological applications. NGT are explicitly mentioned in documents of the BMEL or the FAO [62,63]. In addition, no distinction was made between different NGT technologies e.g., GE approaches mediating small mutations (SDN1/2) or transgenesis (SDN3). This implies, on the one hand, that connecting research results achieved so far with the help of NGT with the defined objectives is still difficult or even impossible. On the other hand, it indicates that almost all policy papers have so far left the choice of means largely open—neither explicitly excluding nor including NGT.

### 2.2. Plant Traits Linked to Sustainable Development Goals

To review the status of (crop) plants developed with NGT against the relatively abstract expectations for NGT application, we translated the latter into plant traits. For this, the documents were screened again for mentioned traits using a lexical search that was iteratively improved (see Methods and Table S2). Overall, it became clear that increasing and securing plant yields were often mentioned in connection with food security and adaptations to climate change. Particularly noteworthy is the point of resilience, which rarely refers to an individually defined property but implies the robustness of crops against several adverse conditions occurring simultaneously, mostly in context with climate change. Consequently, resilience means that plants, usually through a combination of several properties or a superordinate trait [65], are flexible in reacting to a combination of stressors.

The traits most frequently mentioned with 331 and 632 codings (Table 1) relate to yield and resilience, with especially large numbers in peer-reviewed scientific reviews. Even if overarching traits are more concrete compared to the expectations, the term resilience is particularly broad—but it illustrates very well the extensive challenges of plant development from adapting to temperature changes, to water scarcity, to pathogen infestation. Then again, resilience is not always clearly defined and thus generates inaccuracies when compared against actual scientific developments of NGT.

In this regard, to improve the tangibility of the plant traits, the two subordinate and more in-point aspects of resilience, drought tolerance and plant diseases, should be emphasised. Both traits imply yield maintenance at the least but result in an increase if possible. Yield itself is also a very complex trait that is addressed directly by classic breeding and by genetic engineering techniques, for example, for larger or more numerous plant organs, changing the metabolism, or by increasing the efficiency of photosynthesis. Indirectly, yield is connected to drought tolerance, visible in the type of photosynthesis or water use efficiency. However, harvest losses due to pathogen infestation result in reduced yield as well. In addition, both fields were mentioned frequently in our results and therefore represent both important areas in plant research and relevant traits in crop cultivation and breeding. Therefore, to evaluate the research and crop development status in-depth, the traits of drought tolerance and pathogen resistance were selected for a literature review, and the analysis of examples for both resistance against biotic and abiotic stress factors was carried out.

### 2.3. Overview on the Status of Plant Traits Addressed with Genome Editing

Due to the intensive research efforts in recent years, NGT, above all CRISPR/Cas as a genome-editing technique, could be further optimised for use in plants resulting in increasing numbers of applications [14,66]. Before we go into a more detailed description of research for the traits of drought tolerance and pathogen resistance, we shortly present the current state-of-the-art NGT applications in plant development.

In recent scientific literature, a quite comprehensive selection of traits addressed with NGT was already reviewed and compiled [5,13,14,66,67,68,69,70]. We provide an overview in Table S3. Although we consider not only published improvements in scientific literature but commercial developments drawn from the USDA database, the access to knowledge about field trials and developments of NGT plants for commercial purposes is still limited. For one, information of the given traits and modification is often not made available for private property reasons and field trials for commercially developed plants other than in the USA are not, thus far, publicly documented. 

**Figure 2:**
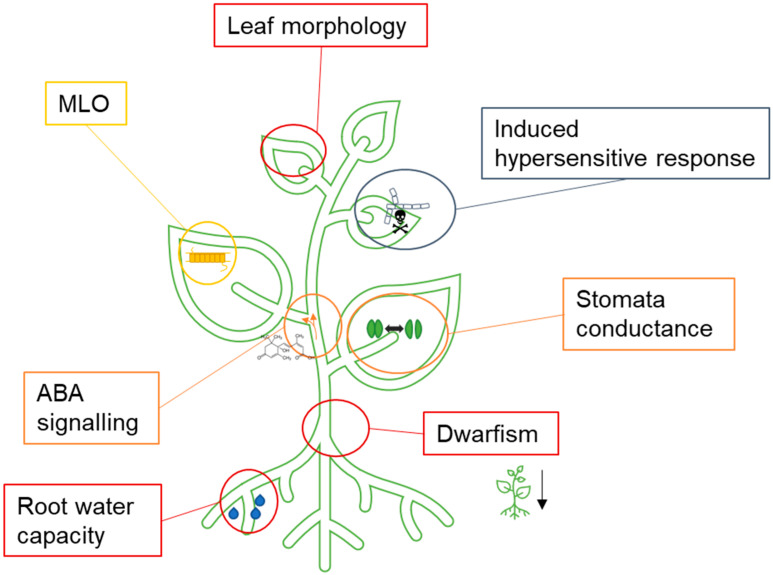
Overview of target points addressed with NGT for the traits of drought tolerance and pathogen resistance. Red, morphological changes from drought tolerance; orange, intracellular changes from drought tolerance; blue, R genes; and yellow, S genes. MLO, Mildew Locus O, ABA, Abscisic acid.

Our results reveal a tendency to improve yield by optimising the relevant plant parts through quality and nutrient contents, especially in the field of fruit, including trees, and vegetable farming (see Table S3) [13,66,67]. Additionally, many staple crops are being developed, such as corn, soybean, potato, rice, or oil crops, such as rapeseed, with traits aiming at an increase in yield, alteration in macronutrient or micronutrient composition, storage and quality properties, simplification of agricultural practices, and abiotic and biotic stress tolerance. The latter two will be addressed in more detail in the following (Figure 2).

### 2.4. Crop Development and Research Status on Drought Tolerance

Due to climate change, it is likely that many regions around the world, including Europe, will experience more frequent dry periods and irregular rainfall [71]. With the plant varieties available today and the current cultivation practices, losses in agriculture are to be expected [72,73,74]—whereby, not only yield, but also quality of the harvest, deteriorates. Generating plants that are adapted to such conditions is a great challenge. Various pathways and physiological properties have been identified to contribute to plant responses connected with elevated drought tolerance. In many cases, however, drought response is reflected in an activated abscisic acid (ABA) metabolism, the accumulation of osmolytes, such as proline, increased antioxidant mechanisms, or the increased expression of chaperones [75,76]. Morphological reactions that are associated with the intracellular stress response but still fulfil complementary functionality include leaf aging, chlorophyll accumulation, changes in the root architecture, or the curling of leaves [77,78]. These mechanisms occur in various combinations depending on the intensity of the stress, the location, the developmental state, and the type of plant.

#### 2.4.1. Possible Setscrews for Adapting to Immediate Drought Tolerance

This section first deals with possible gene locations and molecular targets which have been identified as well as the technical possibilities that are available for the alteration of drought responses. At the molecular level, abiotic stress tolerance can be improved, on the one hand, by the increased regulation or expression of tolerance-mediating genes, e.g., enzymes involved in the regulation of reactive oxygen species, osmolyte accumulation, or cellular homeostasis, on the other hand, it can be improved by changing the so-called sensitivity genes, e.g., transcription factors that control the production of reactive oxygen species [79]. Another essential mechanism for avoiding water loss is the regulation of the stomata aperture, a process that is controlled by ABA [78,80]. It is strongly connected to intrinsic water-use efficiency (WUE) and the corresponding photosynthesis efficiency, especially under water-depriving conditions [81,82]. By increasing stomata and mesophyll conductance, it is possible to engineer the WUE of different plants, though so far only by using classical genetic engineering methods [83,84,85]. In general, genes such as BnaRGA, OsNCED3, or OsABA8ox2 and their respective homologues that are involved in the regulation and biosynthesis of phytohormones, primarily ABA, but also brassinosteroids or gibberellins, are also interesting targets for changing plant drought tolerance beyond the model organism of *Arabidopsis* [65,86,87,88]. In addition, the modification of aquaporins involved in mycorrhiza-associated drought tolerance are individual and potential loci for addressing short-term water stress at the rhizosphere [89,90], and were already shown to increase mesophyll conductance and the intrinsic WUE in rice and tobacco with improved crop performance in field conditions using the classic overexpression analysis [83,85].

Although many theoretical possibilities for optimising drought tolerance based on individual genes are available, it is discussed that actual resilience to shorter periods of drought can only be achieved by adapting multiple genetic locations [91]. Accordingly, drought tolerance is a complex trait—though this assumption is mainly based on differential gene expression comparing drought-stressed plants with control sets. Even if some genes and their role in the plant response to drought stress have been described (and differences in the type and extent of drought stress must also be considered), it might not be necessary to modify all genes that are differentially regulated under drought stress to promote tolerance for a certain drought condition. This is also not the case with conventionally bred plants, where sometimes two or more gene locations in e.g., rice or maize have been changed to improve drought tolerance in the field [92,93]. Superordinate regulatory elements, such as transcription factors, could alter the response to drought stress more comprehensively. Alternatively, there is also the possibility of modifying the sequence of cis-regulatory elements with NGT, which subsequently changes the regulation of several genes involved in the tolerance to drought stress [94,95,96]. This was already demonstrated by modifying a certain recognition sequence of a transcription factor in the corresponding cis-regulatory elements whereby its binding and thus, the transcription of the subsequent gene was influenced [23,57,97,98,99]. By this means, several genes of drought tolerance signalling pathways could be regulated simultaneously without influencing other targets of the transcription factor. Another option could be editing tolerance and sensitivity genes simultaneously using multiplex CRISPR methods [79]. These two approaches could be applied to introduce multigenic traits into plants using NGT, with current examples summarised in Table 2 (basic discoveries in model plants) and Table 3 (applied to crop plants).

#### 2.4.2. Changes Altering Drought Tolerance Implemented with the Help of NGT

Several mentioned setscrews are being targeted and investigated with the help of NGT (Table 2 and Table 3). For instance, intracellular reactions to external stimuli are micro-RNAs regulated by Dicer proteins, whereby special Dicer proteins could be associated with abiotic stress and are now being tested in field trials [100,101]. Other examples are homologous genes of heat shock transcription factors that were analysed for their function with the help of CRISPR/Cas in Arabidopsis and can be specifically switched off simultaneously or individually. Double mutants of HSFA6a and HSFA6b demonstrated a higher tolerance to abiotic stress conditions, which were tested in a laboratory environment for root length and survival rate [105]. Due to the lower accumulation of reactive oxygen species in the roots of the mutants, the authors suspect that the genes play an important role in their homeostasis and therefore are involved in various osmotic stress responses. Further potential loci are regulatory elements or transporters in ABA signal transduction, such as ABA sensitive transcription factors (AtAREB1, TaDREB2), which have already been edited with CRISPR/Cas in Arabidopsis and wheat [22,80,96,114]. AtAREB1 overexpression via a modification of the chromatin status resulted in increased drought tolerance, faster stomatal closure, and a dwarf phenotype [22]. Even if the dwarfism in Arabidopsis, if transferable to crop plants, could be disadvantageous for plant cultivation, e.g., due to lower yield, it must first be reproducible in crops in the field, as does the effect of drought tolerance. For example, dwarfism in wheat or barley can increase the stability of the crops in the field [115].

Other morphological or physiological changes that do not directly reinforce or weaken the stress response (e.g., stomatal movements or osmolyte balance) were already tested in trials in the greenhouse that revealed an increased lateral root growth by knock-out of OsABA8ox2 [88]. Lateral root growth is a morphological property that increases the root surface area for water absorption in the soil and may thus contribute to the plant’s drought tolerance, possibly with greater flexibility to the type of drought [65,116]. Changes in the drought tolerance of crops have already been investigated in tomatoes in greenhouse experiments by determining the survival rate, water capacity and phenotype (SlMAPK3 or SlLBD40 knock-out mutants) [108,110] and in maize (ARGOS8 promoter editing), resulting in better performance in the field [26] (see Table 2). In the latter case, there was a slight increase in the yield under drought stress in the flowering period compared to the wild type—but this effect could not be shown under comparable conditions during grain ripening. This highlights the relevance of the growth period and duration of the drought stress for the breeding of new varieties. The consideration of these factors is essential for the selection of the target genes. In addition, yield stability must be guaranteed even under optimal conditions. This was achieved, for example, by hybridising knock-out lines with increased drought tolerance edited in OsSRL1 and OsSRL2 (see Table 2) and wild-type rice lines under greenhouse conditions [103]. The altered phenotype with half-rolled leaves performed better under drought conditions than wild-type lines.

#### 2.4.3. Future Options

Though several approaches have been taken to improve plant performance under drought stress using NGT, the breeding of plants for this trait is challenging. That is because drought tolerance is environment-dependent, i.e., the interplay of soil, precipitation and environment (microclimate) is decisive [93,117]. Nevertheless, by identifying several loci across populations, the selection of targets can be simplified [118] by sequencing, QTL (quantitative trait loci) mapping, and GWAS (genome wide association mapping), etc. [119,120]. The developmental stages of the plant (during germination, flowering, and fruit ripening), water storage capacity, and foliar transpiration can now be mapped, thus relevant genetic loci or SNPs can be identified for the different aspects of plant traits. This information can be used to introduce changes by NGT in one single plant and to test different modifications in parallel, which may significantly reduce breeding cycles. Examples of specific loci that have been identified in maize with the help of GWAS are a vacuolar transporter (ZmVPP1) or loci that are related to the root length and may contribute to increased drought tolerance [121,122]. In addition, knowledge that has already been generated using classical genetic engineering can be used for genome editing [93]. In the review by Hu and Xiong (2014) [93], tested genes and plants are described and listed in detail, e.g., more drought-tolerant varieties with stable yields have already been found in Arabidopsis. In addition, crops, such as rice or wheat, with targets such as LOS5 (ABA biosynthesis), LEA proteins, or C2H2 zinc finger transcription factors, have been tested in the field, showing at least the same performance as the original variety under optimal conditions [93].

### 2.5. Molecular Mechanisms of Plant Filamentous Pathogen Resistance

Resistance to filamentous plant pathogens can be addressed through various types of mechanisms. These are not mutually exclusive, but usually work independently of one another, e.g., at different points within the plant development, or are combined. A defence mechanism of an infected plant against a pathogen includes at least the recognition of the pathogen, the signal transmission, and the defence of the plant (cell) against the pathogen, while the damage is kept as low as possible [123]. In addition, there can be other signalling pathways that trigger the pathogen response in the whole plant or also warn other plants through, for example, volatile signal molecules. This illustrates how diverse the choice of mechanisms for improving resistance can be for a single plant and a plant population and the possibility of combination. The short-lived nature of various resistances in field shows that this is necessary from the point-of-view of plant cultivation [124].

#### 2.5.1. Different Classes of Resistance-Mediating Gene Loci Distinguished in the Literature

(i) R genes (from resistance) are the most frequently documented form of resistance in literature, which include so-called gene-for-gene resistances, and which therefore usually convey resistance to individual pathogen species or strains [125]. The majority of these resistance cases are characterised by the recognition of a key virulence factor of the pathogen (effector-triggered response) and the mostly complete elimination of the pathogen by the plant [126]. Combating the pathogen involves various signalling pathways that ultimately end in a hypersensitive response at the site of the infection. This leads to selective cell death and, among other effects, the build-up of physical barriers to prevent the pathogen from spreading [127]. This results in a maximum evolutionary pressure on the side of the pathogen and therefore the time to overcome this resistance is often only in the range of 1–5 years [123]. Efforts to find new R genes or new alleles for R genes and to transfer them into crops usually have only a short effect. The advantage of this type of resistance is that it usually only costs the plant limited resources, such as newly formed proteins or the loss of tissue when the pathogen actually infests, thus limiting the potential of yield decrease. 

(ii) S genes (from susceptibility) are plant gene locations that are exploited by the pathogen during colonisation. Their loss, impairment, or loss of function can confer a broader resistance to several pathogens, as a key mechanism for infection is no longer available [124,125,128] The (complete) loss of gene function can lead to decrease in plant viability or crop yield. However, the loss of an attack mechanism cannot be compensated by the pathogen or would have to result in the evolution of a new attack path [128], which is highly unlikely and has not yet been documented for a filamentous pathogen. Thus, impairment of S genes can provide long-term strategies which have been pursued for several years [125]. Efforts to identify new targets are continuously on-going and recently, the use of RNA-seq analysis in existing wheat varieties with differing resistance to wheat yellow rust lead to the discovery of an S gene locus conferring complete resistance (TaBCAT1, [129]).

(iii) Other resistances rely on the level of so-called quantitative resistance, which is usually not pathogen-specific, but acts on the level of signal transduction (from the reception of the pathogen to the physiological response of the plant through hypersensitive response) or structural defence. These resistance genes are sometimes called adult plant resistance genes (APR) or quantitative resistance genes. The build-up of physical barriers after a pathogen attack, e.g., callose deposition at cell boundaries, is part of such a structural defence, which may also be used against a wide variety of pathogens. Such resistances do not confer complete resistance to a certain pathogen and can also be influenced by a wide variety of environmental factors, e.g., a plant that is already affected by abiotic stresses, such as drought or lack of nutrients, might not be able to expend as many resources on structural pathogen defence as a healthy plant [130]. 

Concerning the lifespan of quantitative resistances, it has been demonstrated that, compared to R gene-based resistance, a longer use of this resistance mechanism elapses until it is overcome by the pathogen, as there is not such a great selection pressure on the pathogen [123]. The combination of several (partial) resistances in one plant leads to an increase in the time it takes for the pathogen to overcome resistance and has already been successful in the past [131]. Commercial wheat breading has used APR alleles, such as *Sr2* and *Lr34*, conferring partial resistance against stem rust and powdery mildew, respectively, as early as 1915. Since then, these loci have been genetically mapped and their derived alleles stacked, and the combined use continues until today [131,132]. The research field of quantitative resistance is still wide open and newly researched mechanisms, or more precisely, the desired trait loci, are still continuously identified. Here, too, researchers place their hope in genetic engineering, to rapidly establish polygenetic resistances to maximise the time of the effective period of use until the resistance mechanism is finally overcome [123].

#### 2.5.2. Changes Altering Pathogen Resistance Implemented with the Help of NGT

OsERF922 is an example of a well-studied transcription factor that can be attributed to a quantitative resistance mechanism. It negatively regulates plant defence genes in rice and its knock-out or knock-down confers not only resistance to fungal infection but also enhances salt tolerance [133,134]. While involved in the ABA homeostasis of the plant, this ERF (ethylene responsive factor) seems not to activate classic hormone signalling pathways, i.e., jasmonic acid (JA) and salicylic acid (SA) pathways, that end in a hypersensitive response to confer resistance, such as R genes. An example for an R gene of that mechanism is the WRKY52 from grape that is involved in SA-mediated hypersensitive response and cell death [135]. However, R genes of this class were less abundantly targeted with NGT than genes of the S gene class (see Table 4). 

The potential of NGT is particularly high in this sub-area of resistance to eukaryotic pathogens, as illustrated by the application of NGT from the first establishment of the method in plants to approval of a resistance trait developed in the laboratory (<2 years: TaMLO-A, -B, -D Knockout by TALEN 2014 [47], Approval 2015: USDA 15-238-01). Here, prior knowledge of the relevant genetic locations was used for the introduction of the trait in wheat. The approval of the USDA enables the non-regulated commercial use of this modified plant by the applying company.

Crop plants, with their often polyploid genomes, can be modified much easier with the help of NGT compared to classical genetic engineering. The technique also offers potential to apply the knowledge of the specific resistance loci to other plants through targeted interventions or to bring about the change in the polyploid crop, as happened with the *MLO* locus. Events of where NGT have been used successfully, particularly with regard to the deletion of S genes, e.g., to confer plant resistance to powdery mildew, are listed in Table 4. The resistances obtained make use of the well-known S gene locus *MLO* from barley [142], which, due to its loss, permanently prevents pathogen infection and has thus been mediating resistance for over 20 years in conventional varieties [125]. By identifying and targeting the corresponding homologs, it was possible to establish resistance—most likely equally long-lasting—to powdery mildew in other crops [47,48], as well as proof of concept in apple and grape [143]. Further studies identified natural loss of functional alleles of *MLO* loci in tomato and pea. The simultaneous knock-out of three *MLO* loci in hexaploid wheat achieved by CRISPR/Cas9 should be emphasised here [140]. Other S gene loci were also targeted in rice, tomato, watermelon, and wheat to develop resistance by eliminating the transcription of the functional gene locus [109,133,136,137,138].

The resistance through S genes has so far followed the loss of function of the gene (product) and thus prevents exploitation by the pathogen in the long term. The loss of the gene product in the entire development process of the plant can, but does not have to, lead to losses in terms of crop yields of the respective plant species or variety [144]. One way to circumvent this yield loss can be to prevent the pathogen-specific exploitation of the gene by modifying the corresponding areas of the promoter, demonstrated in rice for the *Xanthomonas oryzae* pv. *oryzae* that caused bacterial blight [145,146]. This way yield loss may be avoided by keeping cellular functions of the gene product in normal metabolism, specifically through targeted interventions that are now made possible by NGT.

## 3. Discussion

### 3.1. Identifying Political, Societal, and Economic Expectations for Use of NGT Plants in Agriculture

#### 3.1.1. Aligning Identified Expectations with Scientific Development

Comparable to the controversy of potential benefits of classic transgenic plants, debate is on-going; its aims can be achieved by application of NGT for crop development, especially in terms of more sustainable agriculture and food systems. This has only recently been displayed by the diverging opinions of diverse stakeholders on the extent of contributions to EU sustainability goals in the study by the European Commission on the status of new genomic techniques [58].

Our analyses revealed that expectations of different stakeholders and groups with political, economic, or scientific backgrounds differ among these groups and mainly refer to social or environmental goals for the near future, in line with the framework of the Sustainable Development Goals. Stakeholder expectations identified in our study remained very general as illustrated by the two most frequently mentioned aims, food security and adaptation to climatic changes. We found that authors of the analysed statements often do not differentiate between the use of NGT and other biotechnologies and typically do not specify how expectations in plant development and agricultural practice could be realised. 

This is comparable with previous studies on expectations regarding transgenic plants in the 1990s, which show that expectations have basically remained the same [147,148]. We assume that these previous statements for the kind and extent of traits to be achieved by the application of transgenic plants have contributed to current expectations. Further, the rapid advancement of NGT tools in research as well as the developments in medical and industrial applications has likely accelerated the stakeholder discussion on near future goals for plant development.

Our results underline that expectations for the development of new crop plants stated in discussions or opinion papers rarely directly manifest as respective traits developed via NGT. It thus remains unclear to what extent NGT-plants can meet unspecified stakeholder expectations. In particular, with the narrow time frames, e.g., by 2030 linked with SDG or the Farm-to-Fork strategy of the EU [55]. Today, only a few NGT-plants have been reported to be available on the market [149] and it remains difficult to estimate which plants will actually reach the market, as certain NGT plants have been cleared from regulation—f.e. by the USDA for the US market - and follow-up reports are not to be expected.

Nevertheless, NGT-plants are currently being developed within in the categories we identified (Table 1), such as biotic and abiotic resistance, yield improvement, or alteration of nutritional content [149,150]. Currently, the highest number of products in advanced development stages are likely those with biotic resistances or modified composition for nutritional and agronomic purposes [149,150]. 

Additional stakeholder interests can be assumed to go beyond the aims occurring in the documents analysed in our study and thus could not be identified with the methodology used. While, e.g., traits with altered nutritional capacity are mainly associated with aims and expectations involving food security and quality in our analyses (Table 1), it has also been discussed that these traits are developed or at least recommended to be developed to promote consumers’ acceptance towards NGT-based products because these products promise to provide individual benefits to consumers [151]. In a similar vein, plants with modified agronomic traits are developed to meet demands of farmers, the food industry for facilitation of agricultural practices, for improved storing, or for processing properties, thereby possibly affecting interest in the application of NGT products from an economical perspective [152].

Several of these aims would further fit within the economic interest, especially of the biotechnology and breeding sectors in the expansion and facilitation of product development based on NGT applications, which have been previously documented [153]. In our study, identified aims and expectations generally tend to derive from the socio-political field and are only loosely connected with economic interests. Expected objectives, such as the promotion of biotechnological industry, are only rarely mentioned. 

#### 3.1.2. Resistance against Fungal Pathogens

Resistance traits against fungal pathogens are considered to be further progressed as compared to the stage of development of most other plant traits addressed by NGT [53,152]. Accordingly, in our results we identified several proof-of-principle studies and development of resistance traits reported to provide pathogen resistance in the laboratory or greenhouse. In contrast, we identified only two publications on field trials. Three NGT plants have been approved for cultivation in the USA [150], but the available data do not allow for conclusions on the actual performance of these and the extent of pathogen resistances.

Resistance against powdery mildew by the *mlo* S-gene alteration, as reviewed here, is often discussed as a prime example. Knock-out or other loss-of-function mutations are technically comparably simple to achieve in short time-frames [48,53] and were mimicked in homologous genes of polyploid species, such as wheat [47]. As the deletion of the S-gene function blocks the entry pathways for pathogens, it is therefore expected to have a longer effective period of use, as exemplarily described for the naturally occurring powdery mildew resistances through *mlo* in some barley varieties [125]. 

It remains to be observed whether simplicity of genetic modifications and long periods of resistance are valid for the development of defence traits against other fungal pathogens, because (i) beneficial genetic variants have to be known, (ii) even exact mimicking of genetic modification known from one species needs testing for each new genetic background, line, or species, and to be newly verified in the field, and (iii) fitness penalties have been reported for *mlo* loss of function mutations in barley [154]. While they have not been reported, e.g., in the case of genome-edited wheat, such tradeoffs are known from other modifications of S-genes, especially in the case of complete knockouts [154]. In general, defence responses may also differ with changing climatic conditions and may lead to diminished pathogen resistance with high temperatures [155,156], stressing the call for more resilient plants.

Few genes have been identified to confer resistance to multiple pathogens in our literature review. It can be assumed that only if resistance against a multitude of fungal pathogens can be achieved in parallel, will applying broad-spectrum fungicides be avoided in conventional farming.

#### 3.1.3. Aiming at Drought Tolerance

Many plant traits, and especially with resilience or tolerance towards abiotic stressors, are genetically, developmentally, and physiologically complex and often composed of different sub-traits [118]. Drought tolerance is likely the most widely investigated example of abiotic traits to be achieved by classical genetic engineering or NGT and the multitude of publications in basic drought research is hardly to be overviewed. Nevertheless, thus far no NGT-based plants with drought tolerance traits are available on the market but two new NGT-based plants, soy bean and maize, have been approved for cultivation in the USA [150]. As depending on the respective regulatory regime (e.g., [157]), a proof of concept or functionality for a trait is usually not required for a GMO authorisation, hence, product performance remains often unclear and its success left to the market.

Despite the long history of drought research, also classic transgenic plants with drought resistance traits are rare on the market—in the period from 2015 to 2019 only three have been newly approved or marketed [150]. Usually, constitutive overexpression of drought-related transcription factors was employed [158,159], with a maximum yield increase of 2–4% reported for field cultivation, which is comparable to conventionally bred varieties [117,160]. This has been discussed to be connected to the redistribution of resources in plant metabolism and subsequent yield loss, mainly in periods without water deficits [117].

To meet the complexity of drought tolerance, not only classic transgenic, but also current NGT approaches target higher-level monogenetic setscrews in plant regulatory networks, usually transcription factors or hormone receptors whose expression is altered; accordingly, downstream responses of the metabolism are changed often to an unknown extent. Approaches to introduce molecular switches or to adapt regulatory regions of downstream genes are, according to our results, rather in the status of general method development, than connected to specific plant traits. This, however, still requires more knowledge about the interplay of individual processes, especially due to the numerous links and overlaps between different signalling pathways and stress responses (e.g., with heat tolerance or oxidative stress).

While the success of traits, such as pathogen resistance, can be easily measured under laboratory conditions, it is not always clear from the literature on drought research when exactly a plant is considered to be drought tolerant or has reached another abiotic tolerance. As drought might occur at different time points and for different periods through the year, drought tolerance and yield cannot be easily related. In this regard, it is important to assess to which extent and in which conditions a crop exhibits tolerance. Thus, it needs to be described how the trait of drought tolerance manifests under defined conditions, be it a general ability of the plant to grow in drier habitats, a development-dependent tolerance, or a resistance in short drought periods. As drought response is highly depending on the environmental habitat, the term “drought tolerance” is likely too broad. The investigation of drought-adapted plants has shown that different strategies of drought tolerance have proven counteractive in different environmental contexts [117,160]. Thus, the development of drought tolerant plants or other abiotic resistances may demand an adaption to local needs even more and therefore require development of several differentially adapted varieties.

## 4. Materials and Methods

### Content Analysis of Political Expectations towards Plant Development

In this study, we used a semi-quantitative content analysis modified from [161,162], which is based on an explorative case study approach commonly used to qualitatively identify factors within a specific universe [162,163,164]. In our case, this universe was selected to be political, supra-national, and scientific institutional papers discussing future strategies of agriculture, including plant breeding, thus following the purposive sampling concept in order to identify relevant perceived factors within this specific group of actors [163]. The two sets of factors we attempted to explore were, first, expectations towards genome editing and, second, corresponding plant traits. 

In order to identify expectations towards new genetic engineering, apart from specialist publications, statements from various interest groups and political documents were researched. This research was carried out with the help of various search engines and databases (Google Scholar (https://scholar.google.de/, accessed on 16 December 2021, Web of Science (https://www.webofknowledge.com/, accessed on 16 December 2021), databased of governmental departments, EU database (https://op.europa.eu/en/web/general-publications/publications accessed on 16 December 2021); FAO database (http://www.fao.org/publications/en/ accessed on 16 December 2021); Ecosia (https://www.ecosia.org/?c=de accessed on 16 December 2021)). Different document categories were identified and classified into (i) political documents from Germany, such as strategy papers from ministries (Federal Ministry for Education and Research, BMBF and Federal Ministry of Food and Agriculture, BMEL) outlining political aims regarding agriculture, (ii) political documents and strategic papers of the European Union, (iii) documents/statements and strategic papers of international organisations, such as UN bodies, such as the Food and Agriculture Organization (FAO) or the Organisation for Economic Co-operation and Development (OECD), documents/statements of scientist organisations and associations, such as the Leopoldina, as well as peer-reviewed scientific reviews. These documents, a total of 63 (see Table S1, Supplementary Methods), were viewed and used for a detailed analysis of properties and general objectives. We employed a deductive coding strategy in an iterative design by developing codes based on assigned categories from the literature in order to identify, summarise, and semi-quantitatively evaluate sections of content within a text [59,60]. The coding was carried out with the help of the MAXQDA program (VERBI Software. (2017). MAXQDA 2018 [computer software]. Berlin, Germany: VERBI Software. Available from https://www.maxqda.com, accessed on 16 December 2021) and was conducted manually.

As a first step, expectations and general objectives in connection with new genetic engineering and plant breeding were extracted from the documents using the framework of the UN Sustainable Development Goals (SDG, Agenda for Sustainable Development; [56]). This framework is especially appropriate for our analysis, as, for one, current political strategies of UN members are entitled to enforce SDG compliance. Secondly, they are applicable to all countries, as by definition, no country has yet achieved comprehensive sustainable development. Thirdly, within this framework, societal, economic and ecologic aims are translated into 17 main goals in order to make these aims more tangible and measurable. To achieve the latter, those 17 goals are further subdivided, which facilitates text analysis with a pre-defined coding scheme. The SDG are a recognised framework for the analysis of goals that has already been frequently applied in the scientific literature and for research purposes, e.g., in connection with the bioeconomy, or used as a basis for studies [165,166,167]. The overarching SDG and, where applicable, the subgoals, were used as deductive codes and analysed. 

Second, to delineate plant traits from those general objectives, the documents were automatically screened (lexical analysis) using search terms selected on the basis of scientific experience and literature [168] (see Table S2) and automatically coded based on this selection. The coding was reviewed and, in the event of duplications or false-positive results (e.g., coding in references), removed or the search terms adjusted. The frequencies of plant traits and objectives were related to one another using code relation matrices of MAXQDA. 

Based on the coding, the most relevant plant traits related to plant breeding objectives were selected for further analysis. To verify our coding and selection approach, the results were revised iteratively. Where applicable, codes were specified or added to the set. 

## 5. Conclusions

The challenges that currently prevent sustainability goals from being achieved are complex and go far beyond the capabilities of a single technology. Nonetheless, SDG require to be fulfilled in a short timeframe and therefore need to be addressed simultaneously. In this regard, the stakes are high for NGT-based plant development. Firstly, complex traits, such as abiotic stress tolerances, need to be understood more comprehensively and subsequently introduced into crop plants. Secondly, the traits must be sustainable in the field under stress and standard conditions; on the one hand, they must increase or at least maintain yield and, on the other hand, they must not have any counter effects or trade-offs within their own or other SDG. More precisely, negative effects on the environment have to be minimised and increased input of fertiliser, water, or pesticides must be avoided. The latter, in particular, is often still unclear, as for a modified plant, be it with a modified fatty acid metabolism or vitamin content, all further challenges for cultivation remain, due to climate change or pest pressure, for example.

A high number of modifications have been developed to alter plant traits and have already been established under lab or green house conditions (Table 4). However, publicly available data on the behaviour of crop varieties in the field and environment are scarce. Therefore, field testing should be carried out to assess whether plants manifest the desired traits and whether there are no adverse effects that contradict sustainable cultivation and environmental protection. Further, complex traits, in particular, that affect the expression of a multitude of genes in plant metabolism or lead to potentially altered fitness, create additional new challenges for the environmental risk assessment [5,169].

Currently, mostly plants with individual new traits are being developed [2]. However, environmental conditions to which crop plants must respond in the future cannot be addressed individually. To meet high expectations, varieties would need to be flexible and resilient to changing environmental conditions and, where possible, combine several new traits. However, for the SDG to be reached, a narrow timeframe of 10 to 20 years is needed. NGT may cut the time to identify the genetic bases and introduce the respective genetic modifications. However, this alone will not provide a solution. New varieties still have to be developed, bred, tested, and established on the market, just as for conventionally bred plants [170].

According to current studies, most new plant varieties will contain biotic resistances or improved nutritional and agronomic properties, but rarely contain abiotic stress tolerances [150]. Apart from the availability on the market, it remains unclear which plant varieties and to what extent they will be grown and thus contribute to sustainability goals in the short and medium term. The choice of the plant variety—conventionally bred or genetically engineered—is in the end, only one measure among many. To be able to respond to the complex overall environmental conditions and to address aims such as adaption to climate change and improved yields in the long run, a variety of agricultural measures will be necessary to increase sustainability and resilience in agriculture [77]. Development of these measures will need comparable attention and research efforts.

## Figures and Tables

**Table 1:** Numbers of plant trait codings in analysed documents and co-occurrence with SDG codings.

	Resilience	Salt Tolerance	Drought Tolerance	Extreme Temperatures	Pathogens	Plant Nutrition	Weed Resistance	Yield	Nutritional Capacity
Total occurrence	632	22	106	51	227	186	134	331	245
Political documents Germany (12)	10	2	17	5	2	69	3	13	14
Political documents EU (6)	29	0	1	0	7	9	7	9	22
Political documents international organisations (10)	190	5	52	19	46	70	31	124	134
Scientist organisations and associations (8)	33	2	14	9	28	7	12	25	22
Peer-reviewed, scientific reviews (27)	370	13	22	18	144	31	81	160	53
SDG 1 no poverty	0	0	0	0	0	0	0	1	1
1.4 equal access to resources	0	0	0	0	0	0	0	1	1
SDG 2 zero hunger	14	1	2	4	5	10	1	19	9
2.1 nutrition quantity and food security	9	1	2	2	3	4	1	17	5
2.3 improve smallholder situation	2	0	0	0	0	0	0	1	3
2.5 ensure agricultural genetic diversity	0	0	0	0	0	0	0	0	0
2.4 sustainable and resilient agriculture	8	0	0	2	4	5	1	7	3
2.2 nutrition improvement	7	1	2	1	3	7	1	10	5
SDG 3 good health and well-being	1	0	0	0	0	2	0	2	2
3.9 reduce illness from contamination/allergy	1	0	0	0	0	2	0	2	2
SDG 7 affordable and clean energy	0	0	0	0	0	0	0	0	0
7.2 renewable energy	0	0	0	0	0	0	0	0	0
SDG 8 decent work and economic growth	0	0	0	0	0	1	0	1	2
8.1 inclusive economic growth	0	0	0	0	0	1	0	1	2
8.5 employment	0	0	0	0	0	0	0	0	0
8.2 innovation	0	0	0	0	0	0	0	0	0
SDG 12 responsible consumption and production	0	0	0	0	0	0	0	0	0
12.5 reduce waste, recycle	0	0	0	0	0	0	0	0	0
SDG 13 climate action	14	3	8	8	4	9	1	12	1
13.2 mitigation actions	0	0	0	0	0	0	0	0	0
13.1 adaptation actions	14	3	8	8	4	9	1	12	1
SDG 14 life below water	0	0	0	0	0	0	0	0	0
14.1 avoid pollution and overnutrition	0	0	0	0	0	0	0	0	0
SDG 15 life on land	3	0	0	0	1	1	0	2	0
15.1 ecosystem conservation	3	0	0	0	1	1	0	2	0
SUM	34	6	16	16	10	22	2	30	6

**Table 2:** Overview of the plants under development with NGT, aiming at characteristics of increased drought tolerance.

Plant	Intended Trait	Loci	Genetic Changes	Method	Development Stage	Reported Effect	References
*Glycine max,* soybean	Drought and salt tolerance	GmDrb2a and GmDrb2b	Knock-out mutations in GmDrb2a and GmDrb2b (SDN1)	CRISPR/Cas	Field trial registered (USDA)	Not described—in Arabidopsis AtDRB2 dependent micro-RNAs are involved in the abiotic stress response	[100,101]; USDA 17-219-01
*Zea mays,* maize	Improved drought tolerance and yield stability	Confidential information deleted	Base editing in not-specified genes (SDN2)	CRISPR/Cas	Field trial registered (USDA)	Not described in detail: plants with improved drought tolerance and yield stability	USDA 20-168-23
*Zea mays,* maize	Improved drought tolerance and corn yield	*Cis*-regulatory region of ARGOS8	Exchange of the promoter (SDN3) -> change in the expression of the transcription factor ARGOS8	CRISPR/Cas	Field trials 2015; 8 locations in the US in total, each with random block design	Increase in grain yield by 2–3% under drought stress at flowering time. No increase (slight decrease 2–3%) under drought stress during grain ripening	[26,102]
*Oryza sativa,* rice	Drought tolerance	OsABA8ox2	Knock-out mutation in OsABA8ox2 (SDN1)	CRISPR/Cas	Crop—greenhouse/lab trial	Improved drought tolerance through increased ABA sensitivity, reduced ABA degradation and vertical root growth	[88]
*Oryza sativa,* rice	Drought tolerance	OsSRL1 and OsSRL2	Knock-out mutation in OsSRL1 and OsSRL2 (SDN1); subsequent hybridisation with wild type	CRISPR/Cas	Crop—greenhouse/lab trial	Increased survival rate under drought stress, but slightly lower yield under unstressed conditions; in hybrid plants with half-rolled leaves the yield was slightly higher than that of wild-type lines	[103]
*Brassica napus,* canola	Drought tolerance	BnaRGA, BnaA6.RGA	Quadruple knock-out mutant of the BnaRGA gene and simple gain-of-function mutant in the BnaA6.RGA gene (SDN1)	CRISPR/Cas	Crop—greenhouse/lab trial	Gain-of-function mutant with increased drought tolerance and higher ABA sensitivity than wild type, quadruple mutant with low drought tolerance	[38,104]

**Table 3:** Overview over basic research on traits conferring increased drought tolerance.

Plant	Intended Trait	Loci	Genetic Changes	Method	Development Stage	Reported Effect	References
Arabidopsis	Drought tolerance	*cis*-regulatory region of AtAREB1	Activation of gene expression through modification of the chromatin status by AtHAT1 (SDN2) in the cis-regulatory region of AtAREB1	CRISPR- dCas9HAT	In model organism	Higher gene expression of AtAREB1; dwarf phenotype; faster stomatal closure and better survival rate under drought stress	[22]
Arabidopsis	Functional analysis under abiotic stress	HSFA6a und HSFA6b	Knock-out mutations in HSFA6a und HSFA6b (SDN1)	CRISPR/Cas	In model organism	Double mutant with abiotic and osmotic stress tolerance	[105]
*Glycine max,* soybean	Functional analysis under abiotic stress	GmMYB118	Knock-out mutation in GmMYB118 (SDN1) and overexpression	CRISPR/Cas and genetic engineering	Crop—greenhouse/lab trial	Reduced tolerance and lower proline and chlorophyll content in the knock-out—improved properties in the overexpression	[106]
*Oryza sativa,* rice	Functional analysis under abiotic stress	OsNCED3	Knock-out mutation in OsNCED3 (SDN1) and overexpression	CRISPR/Cas and genetic engineering	Crop—greenhouse/lab trial	Reduced tolerance to drought, longer growth, more open stomata due to lower ABA levels in the knock-out—improvement compared to wild type in the overexpression	[86]
*Oryza sativa* rice	Functional analysis under abiotic stress	OsDST	Knock-out mutation in OsDST (SDN1)	CRISPR/Cas	Crop—greenhouse/lab trial	Lower stomatal density and improved water balance under drought stress; high salt stress tolerance; no noticeable phenotype under normal conditions	[107]
*Solanum lycopersicum,* tomato	Functional analysis under abiotic stress	SlMAPK3	Knock-out mutation in SlMPAK3 (SDN1)	CRISPR/Cas	Crop—greenhouse/lab trial	Lower drought tolerance and stronger wilt syndrome in knock-out plants	[108]
*Solanum lycopersicum,* tomato	Functional analysis under abiotic stress	SlNPR1	Knock-out mutation in SlNPR1 (SDN1)	CRISPR/Cas	Crop—greenhouse/lab trial	Lower drought tolerance and more open stomata in knock-out plants	[109]
*Solanum lycopersicum,* tomato	Functional analysis under abiotic stress	SlLBD40	Knock-out mutation in SlBD40 (SDN1) & overexpression	CRISPR/Cas and genetic engineering	Crop—greenhouse/lab trial	Improved drought tolerance in knock-out lines due to increased water retention capacity—overexpression led to a lower drought tolerance	[110]
*Cicer arietinum,* chickpea	Functional analysis under drought stress and method	Ca4CL, CaRVE1	Knock-out mutations in Ca4CL, CaRVE1	CRISPR/Cas	Crop—greenhouse/lab trial	Validation of genome-editing method in chickpea using protoplast transfection	[111]
*Triticum aestivum,* wheat	Drought tolerance	TaERF3 and TaDREB2	Knock-out mutations in TaERF3 and TaDREB2 (SDN1)	CRISPR/Cas	Crop—greenhouse/lab trial	DREB2 and ERF3 were identified in wheat and rice as important genes in the drought stress response; in wheat, the expression of TaERF3 and TaDREB2 reacts to drought stress	[112,113,114]

**Table 4:** Overview of the plants under development with NGT aiming at resistance to filamentous pathogens.

Plant	Intended Trait	Loci	Genetic Changes	Method	Development Stage	Reported Effect	References
*Brassica napus,* canola	Fungi pathogen resistance	Confidential information deleted	Confidential information deleted	Gene editing, not specified	Registered for commercialisation	Resistance to fungal pathogens	USDA 20-168-24
*Oryza sativa japonica,* rice	Resistance to rice blast	OsERF922	Knock-out mutation (SDN1)	CRISPR/Cas	Crop—field trial/greenhouse	~50–70% higher resistance	[133]
*Citrullus lanatus,* water melon	Resistance to *Fusarium oxysporum*	ClPSK1	Knock-out mutation (SDN1)	CRISPR/Cas	Crop—greenhouse/lab trial	19–60% higher resistance	[136]
*Solanum lycopersicum,* tomato	Multi-resistance	SlDMR6	Knock-out mutation (SDN1)	CRISPR/Cas	Crop—greenhouse/lab trial	~20% higher resistance to 3 pathogens	[137,138]
*Solanum lycopersicum,* tomato	Resistance to powdery mildew	SlMLO1	Knock-out mutation (SDN1)	CRISPR/Cas	Crop—greenhouse/lab trial	Complete resistance	[48]
*Solanum lycopersicum,* tomato	Resistance to powdery mildew	PMR4	Knock-out mutation (SDN1)	CRISPR/Cas	Crop—greenhouse/lab trial	Higher resistance to powdery mildew	[139]
*Solanum lycopersicum,* tomato	Resistance to Botrytis cinerea	SlNPR1	Knock-out mutation (SDN1)	CRISPR/Cas	Crop—greenhouse/lab trial	33–40% higher resistance	[109]
*Triticum aestivum,* wheat	Resistance to powdery mildew	TaEDR1-A, -B und -D	simultaneous Knock-out in 3 loci (SDN1)	CRISPR/Cas	Crop—greenhouse/lab trial	Reduction of infection by ~50%	[140]
*Triticum aestivum,* wheat	Resistance to powdery mildew	TaMLO-A, -B, D	Knock-out mutation (SDN1)	TALEN	Crop—greenhouse/lab trial, 2 varieties	Complete resistance	[47], USDA 15-238-01
*Vitis vinifera,* grape	Resistance to Botrytis cinerea	VvWRKY52	Knock-out mutation (SDN1)	CRISPR/Cas	Crop—greenhouse/lab trial	50% higher resistance	[141]

## Supplementary Materials

The following are available online at https://www.mdpi.com/article/10.3390/plants11020212/s1, Table S1: Documents used for the analysis of NGT expectations, Table S2: Search terms used for lexical analyses in MAXQDA, Table S3: Overview on traits targeted with NGT in crop plants.
