# Supplementary material for: Genome Editing in Crop Plant Research—Alignment of Expectations and Current Developments
Source: Plants (Basel). 2022 Jan 14;11(2):212. doi: 10.3390/plants11020212 (PMC8778883; doi:10.3390/plants11020212)
Supplement: Supplementary file 1 [file plants-11-00212-s001.zip › plants-1537521-supplementary.pdf]

Supplemental Material

1 Methodological attachment

Table S1 Documents used for the analysis of NGT expectations

Type of document	Number	Reference
Political documents Germany	12	[1-12]
Scientist organisations and associations	8	[13-18] [61-62]
Political documents international organizations	10	[19-28]
Political documents EU	6	[29-33][63]
Peer-reviewed, scientific reviews	27	[34-60]

References

[1] BMWi. Industriestrategie 2030 2019:40.

[2] Bundesministerium für Ernährung und Landwirtschaft (BMEL). Ackerbaustrategie 2035 - Perspektiven für einen produktiven und vielfältigen Pflanzenbau. Berlin: 2019.

[3] Ökologischer Landbau in Deutschland SF 2020. Ökologischer Landbau in Deutschland. BMEL (Bundesministerium Für Ernährung Und Landwirtschaft) 2019:24.

[4] Bundesministerium für Ernährung und Landwirtschaft. Together for a Strong Agriculture Sector - The Bilateral Cooperation Programme of the BMEL 2020:1–15.

[5] Bundesministerium für Ernährung und Landwirtschaft. Perspektive Landwirtschaft - Agrarpolitische Standortbestimmung 2019:1–36.

[6] Maas S, Schmitz PM. Gemeinsame Agrarpolitik der EU. Wirtschaftsdienst 2007;87:94–100. https://doi.org/10.1007/s10273-007-0615-3.

[7] Bundesministerium für Ernährung und Landwirtschaft Strategien für eine nachhaltige Landwirtschaft und Ernährung 2019:1-29

[8] Bundesministerium für Bildung und Forschung (BMBF). Nationale Bioökonomiestrategie 2020:1–68.

[9] Konrad C, Sitta F, Hocker GC, Busen K, Bauer N, Hoffmann C, et al. Antrag an den Deutschen Bundestag Drucksache 19/10166 Chancen neuer Züchtungsmethoden erkennen – Für ein technologieoffenes Gentechnikrecht 2019:1–4.

[10] Ebner H, Künast R, Ostendorff F, Tressel M, Badum L, Gastel M, et al. Deutscher Bundestag Drucksache 19/13072 - Agrarwende statt Gentechnik – Neue Gentechniken im Sinne des Vorsorgeprinzips regulieren und ökologische Landwirtschaft fördern 2019:1–8.

[11] Bundestag D. Anhörung der Deutschen Bundestages - Sachverständige bewerten Auslegung des Gentechnikrechts zwiespältig 2020:1–3.

[12] GIZ. Was ist nachhaltige Landwirtschaft ? Bundesministerium Für Wirtschaftliche Zusammenarbeit Und Entwicklung 2015.

[13] Stephanie Franck (BDP), Joachim Ruwied (DBV), (OVID) JKK von L, Matthias Braun (DIB), Wolfgang Vogel (UFOP), Helmut Schramm (IVA), et al. Verbändestellungnahme zum Urteil des Europäischen Gerichtshofes zu neuen Züchtungsmethoden 2019:1–4.

[14] Bundesverband Deutscher Pflanzenzüchter e.V. Forderungskatalog: Erwartungen an die Politik 2018:1–6.

[15] Nationale Akademie der Wissenschaften Leopoldina, Deutsche Forschungsgemeinschaft, Union der deutschen Akademien der Wissenschaften. Stellungnahme: Wege zu einer wissenschaftlich begründeten, differenzierten Regulierung genomeditierter Pflanzen in der EU. 2019.

[16] Kantar Emnid (a Kantar Group Company). Bevölkerungsumfrage zum Thema Pflanzenzüchtung 2020:1–16.

[17] Bundesverband Deutscher Pflanzenzüchter e.V. Landwirtschaft benötigt Fortschritt Nutzung neuer Züchtungsmethoden muss möglich sein 2018:1–7.

[18] Bundesverband Deutscher Pflanzenzüchter e. V. DIE ZUKUNFT DER LANDWIRTSCHAFT – GEMEINSAME HERAUSFORDERUNG Landwirtschaft 2020:1–8.

[19] Fao. FAO success stories on climate-smart agriculture. Fao 2014:28.

[20] Spielman D, Mayes S, Cook D, Penman D. Summaries of presentations : Opening plenary session and parallel sessions FAO International Symposium on “ The Role of Agricultural Biotechnologies in Sustainable Food Systems and Nutrition ” 2016:1–106.

[21] Food and Agriculture Organization of the United Nations. Key to achieving the 2030 Agenda for Sustainable Development. Food Agric 2016:32.

[22] United Nations. Agriculture development, food security and nutrition Report of the Second Committee (UN_AgriFoddSecurtiyNutrituion, S. 1: 165) a /70/478 2015;22203:1–11.

[23] Friedrichs S, Takasu Y, Kearns P, Dagallier B, Oshima R, Schofield J, et al. Meeting report of the OECD conference on “Genome Editing: Applications in Agriculture—Implications for Health, Environment and Regulation.” vol. 28. Springer International Publishing; 2019.

https://doi.org/10.1007/s11248-019-00154-1.

[24] OECD. Report of the OECD Workshop on Environmental Risk Assessment of Products Derived from New Plant Breeding Techniques. Ser Harmon Regul Overs Biotechnol 2016:85.

[25] Internal Co-ordination Group for Biotechnology of Biotechnology (ICGB) of OECD. Biotechnology update ICGB Newsletter No. 33. 2018.

[26] Shukla-Jones A, Friedrichs S, Winickoff DE. Gene editing in an international context: Scientific, economic and social issues across sectors. OECD Sci Technol Ind Work Pap 2018/04, OECD Publ Paris 2018.

[27] Participants OW. High-Throughput Dna Sequencing in the Safety Assessment of Genetically Engineered Plants: Proceedings of the Oecd Workshop. OECD Environ Heal Saf Publ 2016:1–27.

[28] IUCN. Genetic Frontiers for Conservation: APPENDICES 2019.

[29] European Academies' Science Advisory Council. Genome editing: scientific opportunities, public interests and policy options in the European Union. 2017.

[30] European Academies' Science Advisory Council. Commentary: The regulation of genome-edited plants in the European Union Introduction to new plant breeding techniques 2020:1–8.

[31] European Commission. Directorate-general FS. Ad hoc Stakeholder meeting on new genomic techniques 2020.

[32] European Commission. A sustainable Bioeconomy for Europe: Strengthening the connection between economy, society. COM(2018) 673 final 2018.

[33] On R. European Group on Ethics in Science and New Technologies (EGE) Statement on Gene Editing. Jahrb Für Wiss Und Ethik 2017;21. https://doi.org/10.1515/jwiet-2017-0114.

[34] Zhang Y, Malzahn AA, Sretenovic S, Qi Y. The emerging and uncultivated potential of CRISPR technology in plant science. Nat Plants 2019;5:778–94. https://doi.org/10.1038/s41477-019-0461-5.

[35] Kim J Il, Kim JY. New era of precision plant breeding using genome editing. Plant Biotechnol Rep 2019;13:419–21. https://doi.org/10.1007/s11816-019-00581-w.

[36] Kumar R, Kaur A, Pandey A, Mamrutha HM, Singh GP. CRISPR-based genome editing in wheat: a comprehensive review and future prospects. Mol Biol Rep 2019;46:3557–69. https://doi.org/10.1007/s11033-019-04761-3.

[37] Modrzejewski D, Hartung F, Sprink T, Krause D, Kohl C, Wilhelm R. What is the available evidence for the range of applications of

genome-editing as a new tool for plant trait modification and the potential occurrence of associated off-target effects: A systematic map. Environ Evid 2019;8:1–33. https://doi.org/10.1186/s13750-019-0171-5.

[38] Metje-Sprink J, Menz J, Modrzejewski D, Sprink T. DNA-free genome editing: past, present and future. Front Plant Sci 2019;9:1957.

[39] Martin-Laffon J, Kuntz M, Ricroch AE. Worldwide CRISPR patent landscape shows strong geographical biases. Nat Biotechnol 2019;37:613–20. https://doi.org/10.1038/s41587-019-0138-7.

[40] Hussain Q, Shi J, Scheben A, Zhan J, Wang X, Liu G, et al. Genetic and signalling pathways of dry fruit size: targets for genome editing-based crop improvement. Plant Biotechnol J 2020;18:1124–40. https://doi.org/10.1111/pbi.13318.

[41] Huang S, Weigel D, Beachy RN, Li J. A proposed regulatory framework for genome-edited crops. Nat Genet 2016;48:109.

[42] Barrangou R, Doudna JA. Applications of CRISPR technologies in research and beyond. Nat Biotechnol 2016;34:933–41. https://doi.org/10.1038/nbt.3659.

[43] Jansing J, Schiermeyer A, Schillberg S, Fischer R, Bortesi L. Genome editing in agriculture: Technical and practical considerations. Int J Mol Sci 2019;20. https://doi.org/10.3390/ijms20122888.

[44] Schiemann J, Robienski J, Schleissing S, Spök A, Sprink T, Wilhelm RA. Editorial: Plant Genome Editing – Policies and Governance. Front Plant Sci 2020;11:1–4. https://doi.org/10.3389/fpls.2020.00284.

[45] Alok A, Sandhya D, Jogam P, Rodrigues V, Bhati KK, Sharma H, et al. The Rise of the CRISPR/Cpf1 System for Efficient Genome Editing in Plants. Front Plant Sci 2020;11:1–9. https://doi.org/10.3389/fpls.2020.00264.

[46] El-Mounadi K, Morales-Floriano ML, Garcia-Ruiz H. Principles, Applications, and Biosafety of Plant Genome Editing Using CRISPR-Cas9. Front Plant Sci 2020;11:1–16. https://doi.org/10.3389/fpls.2020.00056.

[47] Sedeek KEM, Mahas A, Mahfouz M. Plant genome engineering for targeted improvement of crop traits. Front Plant Sci 2019;10:1–16. https://doi.org/10.3389/fpls.2019.00114.

[48] Das A, Sharma N, Prasad M. CRISPR/Cas9: A novel weapon in the arsenal to combat plant diseases. Front Plant Sci 2019;9:1–8. https://doi.org/10.3389/fpls.2018.02008.

[49] Chen K, Wang Y, Zhang R, Zhang H, Gao C. CRISPR/Cas Genome Editing and Precision Plant Breeding in Agriculture. Annu Rev Plant Biol 2019;70:667–97. https://doi.org/10.1146/annurev-arplant-050718-100049.

[50] Bortesi L, Fischer R. The CRISPR / Cas9 system for plant genome editing and beyond. Biotechnol Adv 2015;33:41–52.

https://doi.org/10.1016/j.biotechadv.2014.12.006.

[51] Fernie AR, Yan J. De Novo Domestication : An Alternative Route toward New Crops for the Future. Mol Plant 2019;12:615–31. https://doi.org/10.1016/j.molp.2019.03.016.

[52] Mushtaq M, Mukhtar S, Sakina A, Dar AA, Bhat R, Deshmukh R, et al. Tweaking genome-editing approaches for virus interference in crop plants. Plant Physiol Biochem 2020. https://doi.org/10.1016/j.plaphy.2019.12.022.

[53] Yin K, Gao C, Qiu J. Progress and prospects in plant genome editing. Nat Publ Gr 2017;3:1–6. https://doi.org/10.1038/nplants.2017.107.

[54] Belhaj K, Chaparro-garcia A, Kamoun S, Nekrasov V. Plant genome editing made easy : targeted mutagenesis in model and crop plants using the CRISPR / Cas system 2013:1–10.

[55] Pixley K V, Falck-zepeda JB, Giller KE, Glenna LL, Gould F, Mallory-smith CA, et al. Genome Editing , Gene Drives , and Synthetic Biology : Will They Contribute to Disease-Resistant Crops , and Who Will Benefit ? 2019.

[56] Hundleby PAC, Harwood WA. Impacts of the EU GMO regulatory framework for plant genome editing 2019:1–8. https://doi.org/10.1002/fes3.161.

[57] Jouanin A, Boyd L, Visser RGF, Smulders MJM. Development of wheat with hypoimmunogenic gluten obstructed by the gene editing policy in europe. Front Plant Sci 2018;9:1–8. https://doi.org/10.3389/fpls.2018.01523.

[58] Hua K, Zhang J, Botella JR, Ma C, Kong F, Liu B, et al. Perspectives on the Application of Genome-Editing Technologies in Crop Breeding. Mol Plant 2019;12:1047–59. https://doi.org/10.1016/j.molp.2019.06.009.

[59] Bao A, Burritt DJ, Chen H, Zhou X, Cao D, Tran LSP. The CRISPR/Cas9 system and its applications in crop genome editing. Crit Rev Biotechnol 2019;39:321–36. https://doi.org/10.1080/07388551.2018.1554621.

[60] Kumar Y & Jain M. The CRISPR – Cas system for plant genome editing : advances and opportunities 2015;66:47–57. https://doi.org/10.1093/jxb/eru429.

[61] Ethikrat der Max-Planck-Gesellschaft. Diskussionspapier zur wissenschaftlichen Bedeutung der Genom-Editierung und zu den potenziell damit verbundenen ethischen, rechtlichen und gesellschaftlichen Fragen 2019

[62] Bundesverband Deutscher Pflanzenzüchter e.V. Kompaktinformation neue Züchtungsmethoden 2019

[63] EU_Commission High Level Group of Scientific Advisors New techniques in Agricultural Biotechnology Scientific Advice Explanatory Note 02/2017

Table S2: Search terms used for lexical analysis in MAXQDA

	Property	Terms
General attributes	Breeding technologies	breeding [Züchtung][Pflanzenzüchtung]; genome editing [Genomeditierung] [Gentechnik]; Crispr/Cas9; gene scissor [Genschere]; plant biotechnology [Pflanzenbiotechnologie]
	Resilience	adaption [Adaption]; resilience [Resilienz]; adaptation [Anpassungsfähigkeit]; resistance [Widerstandsfähigkeit]; hardiness; robustness [Robustheit]
	Yield	yield [Ertrag]; productivity
	Nutritional capacity	nutrition [Nährstoffe]; flavor [Geschmack]; nutrient [Nährwert]; ingredients [Inhaltsstoffe]; protein; vitamin; "gluten intolerance" [Gluten Intoleranz]; "coeliac disease"; "celiac disease"; intolerance; allergy [Allergie]; allergic
Abiotic factors	Drought tolerance	"water stress" [Trockenstress]; "drought tolerance" [Trockentoleranz]; "drought resistance" [Trockenresistenz]; " "drought resilience"; drought [Dürre] [Trockenheit]
	Extreme temperatures	"heat stress"[Hitzestress]; "heat tolerance" [Hitzetoleranz]; "heat re-sistance" [Hitzeresistenz]; "heat resilience"; warming [Erwärmung]; "global warming" [Klimaerwärmung]; "cold tolerance" [Kältetoleranz]; [Frosthärte]; "cold resistance" [Kälteresistenz]; "cold resilience"; "cold stress"[Kältestress]; cold[Kälte]; heat [Hitze]; "extreme temperatures" ["extreme Temperaturen"]; ["extreme Temperaturunterschiede"]
	Plant nutrition	fertilizer [Dünger]; nitrogen [Nitrat];[Nitrit]; [Stickstoff]; phosphate [Phosphat]; nutrient [Nährstoff]
	Salt tolerance	"marginal soils" [nährstoffarm]; "salt tolerance" [Salztoleranz]; "salt re-sistance [Salzresistenz]; "salty soil" ["salzige Böden"]; "salt stress" [Salz-stress]; salinity
biotic	pathogens	disease [Krankheit]; pathogen [Pathogen]; pest [Pest]; [Seuche]; [Pflanzenkrankheiten]; „pesticide tolerance" [Pestizidtoleranz]
	Weed resistance	herbicide [Herbizid]; weed [Unkraut]

Table S3: Overview on traits targeted with NGTs in crop plants

**Superordinate trait**	**Target trait**	**Plant species**	**comments**	**references**

Yield improvement	Improvement and timepoint alteration of fruit development	soybean (*Glycine max*); strawberry (*Fragaria × ananassa)*	SDN1; USDA documents highlight phenotype only	(Cai et al., 2018); (USDA 17-004-01; USDA 20-147-01; USDA 20-168-05)
	Morphological alterations of plant organs	canola (*Brassica napus);* soybean (*G. max*); potato (*Solanum tuberosum*), rice (*Oryza sativa*), corn (*Zea mays*)	SDN1; corn: *waxy* was changed and hybrids showed improved yield (25locations, USA); rice: up to 30% more yield (1000 grain weight per panicle; in field april-september); potato, USDA documents highlight phenotype only	(Zheng et al., 2020; al Amin et al., 2019; Gao et al., 2020; Yang et al., 2018), (USDA 20-168-03;))
macronutrients	Change in oil composition	Pennycress (*Thlaspi arvense)*, millet (*Panicum virgatum),* camelina (*Camelina sativa*), canola (*B. napus*), soybean (*G. max*)	SDN1; USDA documents partly inform about loci (Gm*DGAT1B)*	(USDA 20-066-01; USDA 20-168-14; USDA 20-163-02; USDA 19-189-0; USDA 20-062-04; (Morineau et al., 2017)
	Starch and lignin	corn (*Z. mays*), potato (S. *tuberosum*); alfalfa (*Medicago sativa*)	SDN1; Andersson et al. used protoplasts; USDA see above	(Andersson et al., 2018); USDA 15-352-01; USDA 17-038-02
micronutrients	Allergens	wheat (*Triticum aestivum*)	Lowered gluten content proven; USDA see above	(Sánchez-León et al., 2018) (USDA 17-038-01)
	Micro elements	rice (*O. sativa*), barley (*Hordeum vulgare*)	lowered cadmium accumulation without yield reduction; mutations in OsNramp5; alteration in phosphate content; HvPAPhy_a (Holme et al., 2017)	(Tang et al., 2017; Holme et al., 2017)

	Secondary metabolites	Tomato (*Solanum lycopersicum*); pea (*Pisum sativum*); mustard (*Brassica juncacea*); petunia (*Petunia ×hybrida*)	SDN1; γ-aminobutyric acid- (GABA) (USDA document with much detail) und lycopen content, Multiplex-CRISPR); taste alteration in pea and mustard unclear	(Li et al., 2018); (USDA 20-140-01; USDA 20-168-06; USDA 20-108-01; USDA 20-168-07)
	Reduction of undesirable compounds	potato (*S. tuberosum*); tobacco (*Nicotiana tabacum*);	Reduction of glyalkaloids unclear; nicotine alteration through mutagenesis of *Berberine Bridge Enzyme-Like* (BBL) gene	(USDA 20-168-06; USDA 17-126-01)
Storage and quality properties	Reduced browning	potato (*S. tuberosum*); avocado (*Persea americana*);	SDN1; traits are known: reduced polyphenoloxidase (PPO) activity	(USDA 16-090-01; USDA 20-168-35); (Toledo and Aguirre, 2017; González et al., 2020);
	Quality	tomato (*S. lycopersiucm*)	SDN1; improved fruit detachment (Jointless2; SlMBP21), optimization of plants for Urban Farming (Gene: SELF-PRUNING (SP), SELF-PRUNING-5G (SP5G) and ERECTA (SlER); Parthenocarpie (seedless fruits; Knock-out of SlAGL6 or SlIAA9)	(USDA 20-168-33; USDA 19-338-01; USDA 18-051-01); (Roldan et al., 2017; Klap et al., 2017; Ueta et al., 2017; Jouanin et al., 2018)
Farming improvements	Reduced seed loss	canola (*B. napus*);	unclear	(USDA 20-160-01); (USDA 18-348-01; USDA 18-351-01)
	Glyphosate tolarance	rice (*O. sativa*); flax (*Linum usitatissimum*); water melon (*Citrullus lanatus*)	USDA documents: gene clear, rest unclear; watermelon CRISPR-mediated base editing; rice: combination of CRISPR & non-homologous end joining	(USDA 18-348-01; USDA 18-351-01); (Li et al., 2016; Tian et al., 2018)
Abiotic stress tolerance	Osmotic stress	rice (*O. sativa*); tomato (*S. lycopersicum*);	SDN1; OsDREB1A and OsRR22; in tomato slightly reduced growth	(USDA 17-286-01); (Zhang et al., 2019; Bouzroud et al., 2020)

	Cold tolerance	rice (O. *sativa*)	Higher yield and improved drought tolerances by SDN1 mutation of OsPIN5b, GS3 and OsMYB30	(Zeng et al., 2020)
Biotic stress resistance	Nematode resistance	Soybean (*G. max*)	SDN1; unclear	(USDA 19-281-02)
	Bacteria resistance	Rice (*O. sativa*); orange (*Citrus sinensis* Osbeck); grapefruit (*Citrus* × *paradisi*)	Broad spectrum resistance against wilt (*Xanthomonas ssp.*) by mutagenesis of several *SWEET* genes via TALEN or CRISPR/Cas; Citrus cancer resistance against *Xanthomonas citri subsp. citri (Xcc*) by mutation of CsWRKY22; *CsLOB1* gene and promotor	(USDA 20-143-01) (Oliva et al., 2019; Blanvillain-Baufumé et al., 2017; Varshney et al., 2019; Peng et al., 2017; Jia et al., 2019; Wang et al., 2019; Jia et al., 2017)
	Virus resistance	Tomato (*S. lycopersicum*); cassava (*Manihot esculenta*)	SDN1; improved resistance	(Modrzejewski et al., 2019; Tashkandi et al., 2018; Gomez et al., 2019)

Additional References for Supplemental Table S2 and S3.

USDA 15-238-01 2015 United States Department of Agriculture (USDA). https://www.aphis.usda.gov/biotechnology/downloads/reg_loi/15-238-01_air_inquiry_cbidel.pdf Accessed 17 Nov 2020

USDA 15-352-01 United States Department of Agriculture (USDA). 2015 https://www.aphis.usda.gov/biotechnology/downloads/reg_loi/15-352-01_air_inquiry_cbidel.pdf Accessed 25 Aug 2020.

USDA 15-352-02 United States Department of Agriculture (USDA). 2015 https://www.aphis.usda.gov/biotechnology/downloads/reg_loi/15-352-02_air_response_signed.pdf Accessed 25 Aug 2020.

USDA 16-090-01 United States Department of Agriculture (USDA). 2016 https://www.aphis.usda.gov/biotechnology/downloads/reg_loi/16-090-01_air_inquiry_cbidel.pdf Accessed 25 Aug 2020.

USDA 17-004-01 United States Department of Agriculture (USDA). 2017 https://www.aphis.usda.gov/biotechnology/downloads/reg_loi/17-004-01_air_response_signed.pdf Accessed 31 Sep 2020.

USDA 17-038-01 2017 United States Department of Agriculture (USDA). 2017. https://www.aphis.usda.gov/biotechnology/downloads/reg_loi/17-038-01_air_inquiry_cbidel.pdf. Accessed 25 Aug 2020.

USDA 17-038-02 2017 United States Department of Agriculture (USDA). https://www.aphis.usda.gov/biotechnology/downloads/reg_loi/17-038-02_air_inquiry_cbidel.pdf Accessed 25 Aug 2020

USDA 17-126-01 2017 United States Department of Agriculture (USDA). https://www.aphis.usda.gov/biotechnology/downloads/reg_loi/17-126-01_air_inquiry.pdf Accessed 25 Aug 2020

USDA 17-219-01 United States Department of Agriculture (USDA). 2017. https ://www.aphis .usda.gov/biote chnol ogy/downl oads/reg_loi/17-219-01_air_inquiry.pdf. Accessed 25 Aug 2020.

USDA 17-286-0 2017 United States Department of Agriculture (USDA). https://www.aphis.usda.gov/biotechnology/downloads/reg_loi/17-038-01_air_response_signed.pdf Accessed 25 Aug 2020

USDA 18-051-01 2018 United States Department of Agriculture (USDA). https://www.aphis.usda.gov/biotechnology/downloads/reg_loi/18-051-01_air_response_signed.pdf Accessed 25 Aug 2020

USDA 18-348-01 2018 United States Department of Agriculture (USDA). https://www.aphis.usda.gov/biotechnology/downloads/reg_loi/18-348-01_air_response_signed.pdf Accessed 25 Aug 2020

USDA 18-351-01 2018 United States Department of Agriculture (USDA). https://www.aphis.usda.gov/biotechnology/downloads/reg_loi/18-351-01_air_response_signed.pdfAccessed 25 Aug 2020

USDA 19-189-0 2019 United States Department of Agriculture (USDA). https://www.aphis.usda.gov/aphis/ourfocus/biotechnology/am-i-regulated/regulated_article_letters_of_inquiry/regulated_article_letters_of_inquiry Accessed 25 Aug 2020

USDA 19-266-01 2019 United States Department of Agriculture (USDA). (https://www.aphis.usda.gov/biotechnology/downloads/reg_loi/19-266-01-air-inquiry-

cbidel-a3.pdf Accessed 30 Sep 2020

USDA 19-281-02 2019 United States Department of Agriculture (USDA). https://www.aphis.usda.gov/aphis/ourfocus/biotechnology/am-i-regulated/regulated_article_letters_of_inquiry/regulated_article_letters_of_inquiry Accessed 25 Aug 2020

USDA 20-062-04 2020 United States Department of Agriculture (USDA). https://www.aphis.usda.gov/aphis/ourfocus/biotechnology/am-i-regulated/regulated_article_letters_of_inquiry/regulated_article_letters_of_inquiry Accessed 25 Aug 2020

USDA 20-066-0 2020 United States Department of Agriculture (USDA). https://www.aphis.usda.gov/aphis/ourfocus/biotechnology/am-i-regulated/regulated_article_letters_of_inquiry/regulated_article_letters_of_inquiry Accessed 25 Aug 2020

USDA 20-108-01 2020 United States Department of Agriculture (USDA). https://www.aphis.usda.gov/aphis/ourfocus/biotechnology/am-i-regulated/regulated_article_letters_of_inquiry/regulated_article_letters_of_inquiry Accessed 25 Aug 2020

USDA 20-140-01 2020 United States Department of Agriculture (USDA). https://www.aphis.usda.gov/aphis/ourfocus/biotechnology/am-i-regulated/regulated_article_letters_of_inquiry/regulated_article_letters_of_inquiry Accessed 25 Aug 2020

USDA 20-149-01 2020 United States Department of Agriculture (USDA). https://www.aphis.usda.gov/aphis/ourfocus/biotechnology/am-i-regulated/regulated_article_letters_of_inquiry/regulated_article_letters_of_inquiry Accessed 25 Aug 2020

USDA 20-147-01 2020 United States Department of Agriculture (USDA). https://www.aphis.usda.gov/aphis/ourfocus/biotechnology/am-i-regulated/regulated_article_letters_of_inquiry/regulated_article_letters_of_inquiry Accessed 25 Aug 2020

USDA 20-160-01 2020 United States Department of Agriculture (USDA). https://www.aphis.usda.gov/aphis/ourfocus/biotechnology/am-i-regulated/regulated_article_letters_of_inquiry/regulated_article_letters_of_inquiry Accessed 25 Aug 2020

USDA 20-163-02 2020 United States Department of Agriculture (USDA). https://www.aphis.usda.gov/aphis/ourfocus/biotechnology/am-i-regulated/regulated_article_letters_of_inquiry/regulated_article_letters_of_inquiry Accessed 25 Aug 2020

USDA 20-168-03 2020 United States Department of Agriculture (USDA). https://www.aphis.usda.gov/aphis/ourfocus/biotechnology/am-i-regulated/regulated_article_letters_of_inquiry/regulated_article_letters_of_inquiry Accessed 25 Aug 2020

USDA 20-168-05 2020 United States Department of Agriculture (USDA). https://www.aphis.usda.gov/aphis/ourfocus/biotechnology/am-i-regulated/regulated_article_letters_of_inquiry/regulated_article_letters_of_inquiry Accessed 25 Aug 2020

USDA 20-168-06 2020 United States Department of Agriculture (USDA). https://www.aphis.usda.gov/aphis/ourfocus/biotechnology/am-i-regulated/regulated_article_letters_of_inquiry/regulated_article_letters_of_inquiry Accessed 25 Aug 2020

USDA 20-168-07 2020 United States Department of Agriculture (USDA). https://www.aphis.usda.gov/aphis/ourfocus/biotechnology/am-i-regulated/regulated_article_letters_of_inquiry/regulated_article_letters_of_inquiry Accessed 25 Aug 2020

USDA 20-168-14 2020 United States Department of Agriculture (USDA). https://www.aphis.usda.gov/aphis/ourfocus/biotechnology/am-i-regulated/regulated_article_letters_of_inquiry/regulated_article_letters_of_inquiry Accessed 25 Aug 2020

USDA 20-168-332020 United States Department of Agriculture (USDA). https://www.aphis.usda.gov/aphis/ourfocus/biotechnology/am-i-regulated/regulated_article_letters_of_inquiry/regulated_article_letters_of_inquiry Accessed 25 Aug 2020

USDA 20-168-35 2020 United States Department of Agriculture (USDA).

https://www.aphis.usda.gov/aphis/ourfocus/biotechnology/am-i-regulated/regulated_article_letters_of_inquiry/regulated_article_letters_of_inquiry Accessed 25 Aug 2020

IPCC 2019 Mbow, C., C. Rosenzweig, L.G. Barioni, T.G. Benton, M. Herrero, M. Krishnapillai, E. Liwenga, P. Pradhan, M.G. Rivera-Ferre, T. Sapkota, F.N. Tubiello, Y. Xu, 2019: Food Security. In: Climate Change and Land: an IPCC special report on climate change, desertification, land degradation, sustainable land management, food security, and greenhouse gas fluxes in terrestrial ecosystems [P.R. Shukla, J. Skea, E. Calvo Buendia, V. Masson-Delmotte, H.-O. Pörtner, D.C. Roberts, P. Zhai, R. Slade, S. Connors, R. van Diemen, M. Ferrat, E. Haughey, S. Luz, S. Neogi, M. Pathak, J. Petzold, J. Portugal Pereira, P. Vyas, E. Huntley, K. Kissick, M. Belkacemi, J. Malley, (eds.)]. In press

al Amin, N., Ahmad, N., Wu, N., Pu, X., Ma, T., Du, Y., Bo, X., Wang, N., Sharif, R., and Wang, P. (2019). CRISPR-Cas9 mediated targeted disruption of FAD2–2 microsomal omega-6 desaturase in soybean (Glycine max.L). BMC Biotechnol. **19**: 9.

Andersson, M., Turesson, H., Olsson, N., Fält, A., Ohlsson, P., Gonzalez, M.N., Samuelsson, M., and Hofvander, P. (2018). Genome editing in potato via CRISPR-Cas9 ribonucleoprotein delivery. Physiol. Plant. **164**: 378–384.

Blanvillain-Baufumé, S., Reschke, M., Solé, M., Auguy, F., Doucoure, H., Szurek, B., Meynard, D., Portefaix, M., Cunnac, S., and Guiderdoni, E. (2017). Targeted promoter editing for rice resistance to Xanthomonas oryzae pv. oryzae reveals differential activities for SWEET 14-inducing TAL effectors. Plant Biotechnol. J. **15**: 306–317.

Bouzroud, S., Gasparini, K., Hu, G., Barbosa, M.A.M., Rosa, B.L., Fahr, M., Bendaou, N., Bouzayen, M., Zsögön, A., and Smouni, A. (2020). Down Regulation and Loss of Auxin Response Factor 4 Function Using CRISPR/Cas9 Alters Plant Growth, Stomatal Function and Improves Tomato Tolerance to Salinity and Osmotic Stress. Genes (Basel). **11**: 272.

Cai, Y., Chen, L., Liu, X., Guo, C., Sun, S., Wu, C., Jiang, B., Han, T., and Hou, W. (2018). CRISPR/Cas9-mediated targeted mutagenesis of GmFT2a delays flowering time in soya bean. Plant Biotechnol. J. **16**: 176–185.

Gao, H., Gadlage, M.J., Lafitte, H.R., Lenderts, B., Yang, M., Schroder, M., Farrell, J., Snopek, K., Peterson, D., and Feigenbutz, L. (2020). Superior field performance of waxy corn engineered using CRISPR–Cas9. Nat. Biotechnol. **38**: 579–581.

Gomez, M.A., Lin, Z.D., Moll, T., Chauhan, R.D., Hayden, L., Renninger, K., Beyene, G., Taylor, N.J., Carrington, J.C., Staskawicz, B.J., and Bart, R.S. (2019). Simultaneous CRISPR/Cas9-mediated editing of cassava eIF4E isoforms nCBP-1 and nCBP-2 reduces cassava brown streak disease symptom severity and incidence. Plant Biotechnol. J. **17**: 421–434.

González, M.N., Massa, G.A., Andersson, M., Turesson, H., Olsson, N., Fält, A.-S., Storani, L., Décima Oneto, C.A., Hofvander, P., and Feingold, S.E. (2020). Reduced enzymatic browning in potato tubers by specific editing of a Polyphenol Oxidase gene via Ribonucleoprotein complexes delivery of the CRISPR/Cas9 system. Front. Plant Sci. **10**: 1649.

Holme, I.B., Wendt, T., Gil-Humanes, J., Deleuran, L.C., Starker, C.G., Voytas, D.F., and Brinch-Pedersen, H. (2017). Evaluation of the mature grain phytase candidate HvPAPhy_a gene in barley (Hordeum vulgare L.) using CRISPR/Cas9 and TALENs. Plant Mol. Biol. **95**: 111–121.

Jia, H., Orbović, V., and Wang, N. (2019). CRISPR-LbCas12a-mediated modification of citrus. Plant Biotechnol. J. **17**: 1928–1937.

Jia, H., Zhang, Y., Orbović, V., Xu, J., White, F.F., Jones, J.B., and Wang, N. (2017).

Genome editing of the disease susceptibility gene CsLOB1 in citrus confers resistance to citrus canker. Plant Biotechnol. J. **15**: 817–823.

Jouanin, A., Boyd, L., Visser, R.G.F., and Smulders, M.J.M. (2018). Development of wheat with hypoimmunogenic gluten obstructed by the gene editing policy in europe. Front. Plant Sci. **9**: 1–8.

Klap, C., Yeshayahou, E., Bolger, A.M., Arazi, T., Gupta, S.K., Shabtai, S., Usadel, B., Salts, Y., and Barg, R. (2017). Tomato facultative parthenocarpy results from Sl AGAMOUS-LIKE 6 loss of function. Plant Biotechnol. J. **15**: 634–647.

Li, J., Meng, X., Zong, Y., Chen, K., Zhang, H., Liu, J., Li, J., and Gao, C. (2016). Gene replacements and insertions in rice by intron targeting using CRISPR–Cas9. Nat. plants **2**: 1–6.

Li, X., Wang, Y., Chen, S., Tian, H., Fu, D., Zhu, B., Luo, Y., and Zhu, H. (2018). Lycopene is enriched in tomato fruit by CRISPR/Cas9-mediated multiplex genome editing. Front. Plant Sci. **9**: 559.

Modrzejewski, D., Hartung, F., Sprink, T., Krause, D., Kohl, C., and Wilhelm, R. (2019). What is the available evidence for the range of applications of genome-editing as a new tool for plant trait modification and the potential occurrence of associated off-target effects: A systematic map. Environ. Evid. **8**: 1–33.

Morineau, C., Bellec, Y., Tellier, F., Gissot, L., Kelemen, Z., Nogué, F., and Faure, J. (2017). Selective gene dosage by CRISPR-Cas9 genome editing in hexaploid Camelina sativa. Plant Biotechnol. J. **15**: 729–739.

Oliva, R., Ji, C., Atienza-Grande, G., Huguet-Tapia, J.C., Perez-Quintero, A., Li, T., Eom, J.-S., Li, C., Nguyen, H., and Liu, B. (2019). Broad-spectrum resistance to bacterial blight in rice using genome editing. Nat. Biotechnol. **37**: 1344–1350.

Peng, A., Chen, S., Lei, T., Xu, L., He, Y., Wu, L., Yao, L., and Zou, X. (2017). Engineering canker-resistant plants through CRISPR/Cas9-targeted editing of the susceptibility gene Cs LOB 1 promoter in citrus. Plant Biotechnol. J. **15**: 1509–1519.

Roldan, M.V.G., Périlleux, C., Morin, H., Huerga-Fernandez, S., Latrasse, D., Benhamed, M., and Bendahmane, A. (2017). Natural and induced loss of function mutations in SlMBP21 MADS-box gene led to jointless-2 phenotype in tomato. Sci. Rep. **7**: 1–10.

Sánchez-León, S., Gil-Humanes, J., Ozuna, C. V, Giménez, M.J., Sousa, C., Voytas, D.F., and Barro, F. (2018). Low-gluten, nontransgenic wheat engineered with CRISPR/Cas9. Plant Biotechnol. J. **16**: 902–910.

Tang, L. et al. (2017). Knockout of OsNramp5 using the CRISPR/Cas9 system produces low Cd-accumulating indica rice without compromising yield. Sci. Rep. **7**: 14438.

Tashkandi, M., Ali, Z., Aljedaani, F., Shami, A., and Mahfouz, M.M. (2018). Engineering resistance against Tomato yellow leaf curl virus via the CRISPR/Cas9 system in tomato. Plant Signal. Behav. **13**: e1525996.

Tian, S., Jiang, L., Cui, X., Zhang, J., Guo, S., Li, M., Zhang, H., Ren, Y., Gong, G., and Zong, M. (2018). Engineering herbicide-resistant watermelon variety through CRISPR/Cas9-mediated base-editing. Plant Cell Rep. **37**: 1353–1356.

Toledo, L. and Aguirre, C. (2017). Enzymatic browning in avocado (Persea americana) revisited: History, advances, and future perspectives. Crit. Rev. Food Sci. Nutr. **57**: 3860–3872.

Ueta, R., Abe, C., Watanabe, T., Sugano, S.S., Ishihara, R., Ezura, H., Osakabe, Y., and Osakabe, K. (2017). Rapid breeding of parthenocarpic tomato plants using CRISPR/Cas9. Sci. Rep. **7**: 507.

Varshney, R.K., Godwin, I.D., Mohapatra, T., Jones, J.D.G., and McCouch, S.R. (2019). A SWEET solution to rice blight. Nat. Biotechnol. **37**: 1280–1282.

Wang, L., Chen, S., Peng, A., Xie, Z., He, Y., and Zou, X. (2019). CRISPR/Cas9-mediated editing of CsWRKY22 reduces susceptibility to Xanthomonas citri subsp. citri in Wanjincheng orange (Citrus sinensis (L.) Osbeck). Plant Biotechnol. Rep. **13**: 501–510.

Yang, Y., Zhu, K., Li, H., Han, S., Meng, Q., Khan, S.U., Fan, C., Xie, K., and Zhou, Y. (2018). Precise editing of CLAVATA genes in Brassica napus L. regulates multilocular silique development. Plant Biotechnol. J. **16**: 1322–1335.

Zeng, Y., Wen, J., Zhao, W., Wang, Q., and Huang, W. (2020). Rational Improvement of Rice Yield and Cold Tolerance by Editing the Three Genes OsPIN5b, GS3, and OsMYB30 With the CRISPR–Cas9 System. Front. Plant Sci. **10**: 1663.

Zhang, A., Liu, Y., Wang, F., Li, T., Chen, Z., Kong, D., Bi, J., Zhang, F., Luo, X., and Wang, J. (2019). Enhanced rice salinity tolerance via CRISPR/Cas9-targeted mutagenesis of the OsRR22 gene. Mol. Breed. **39**: 47.

Zheng, M., Zhang, L., Tang, M., Liu, J., Liu, H., Yang, H., Fan, S., Terzaghi, W., Wang, H., and Hua, W. (2020). Knockout of two Bna MAX 1 homologs by CRISPR/Cas9-targeted mutagenesis improves plant architecture and increases yield in rapeseed (Brassica napus L.). Plant Biotechnol. J. **18**: 644–654.
